# MicroRNA-128 suppresses paclitaxel-resistant lung cancer by inhibiting MUC1-C and BMI-1 in cancer stem cells

**DOI:** 10.18632/oncotarget.22818

**Published:** 2017-11-30

**Authors:** Hyebin Koh, Hyeri Park, Nisansala Chandimali, Do Luong Huynh, Jiao Jiao Zhang, Mrinmoy Ghosh, Meeta Gera, Nameun Kim, Yesol Bak, Do-Young Yoon, Yang Ho Park, Taeho Kwon, Dong Kee Jeong

**Affiliations:** ^1^ Laboratory of Animal Genetic Engineering and Stem Cell Biology, Department of Animal Biotechnology, Faculty of Biotechnology, Jeju National University, Jeju, Republic of Korea; ^2^ Department of Bioscience and Biotechnology, Bio/Molecular Informatics Center, Konkuk University, Seoul, Republic of Korea; ^3^ BRM Institute, Seoul, Republic of Korea; ^4^ Laboratory of Animal Genetic Engineering and Stem Cell Biology, Subtropical/Tropical Organism Gene Bank, Jeju National University, Jeju, Republic of Korea

**Keywords:** cancer stem cells, Muc1-C, BMI-1, microRNA-128, paclitaxel-resistant

## Abstract

The existence of cancer stem cells (CSCs) is the main reason for failure of cancer treatment caused by drug resistance. Therefore, eradicating cancers by targeting CSCs remains a significant challenge. In the present study, because of the important role of BMI-1 proto-oncogene, polycomb ring finger (BMI-1) and C-terminal Mucin1 (MUC1-C) in tumor growth and maintenance of CSCs, we aimed to confirm that microRNA miR-128, as an inhibitor of BMI-1 and MUC1-C, could effectively suppress paclitaxel (PTX)-resistant lung cancer stem cells. We showed that CSCs have significantly higher expression levels of BMI-1, MUC1-C, stemness proteins, signaling factors, and higher malignancy compared with normal tumor cells. After transfection with miR-128, the BMI-1 and MUC1-C levels in CSCs were suppressed. When miR-128 was stably expressed in PTX-resistant lung cancer stem cells, the cells showed decreased proliferation, metastasis, self-renewal, migration, invasive ability, clonogenicity, and tumorigenicity *in vitro* and *in vivo* and increased apoptosis compared with miR-NC (negative control) CSCs. Furthermore, miR-128 effectively decreased the levels of β-catenin and intracellular signaling pathway-related factors in CSCs. MiR-128 also decreased the luciferase activity of MUC1 reporter constructs and reduced the levels of transmembrane MUC1-C and BMI-1. These results suggested miR-128 as an attractive therapeutic strategy for PTX-resistant lung cancer via inhibition of BMI-1 and MUC1-C.

## INTRODUCTION

Lung cancers cause 30% of cancer-related deaths worldwide [1]. Lung cancer is categorized into small cell lung cancer (SCLC) and non-small cell lung cancer (NSCLC). NSCLC represents 70–80% of all lung cancer cases and includes squamous cell carcinoma, large cell carcinoma, and adenocarcinoma [2, 3]. To date, common therapies used in cancer treatment are surgery, chemotherapy, radiotherapy, targeted therapy, or a combination of treatments. When a diagnosis is made, up to 70% of patients with lung cancer appear as local or disseminated disease. Chemotherapy is useful for patients with local or metastatic disease to give temporary relief [4], and paclitaxel-based chemotherapy is a standard first-line treatment for NSCLC patients [5]. However, during therapy, treatment often fails because the cancer develops drug resistance [6]. The development of chemo-resistance is a frequent problem during therapy for locally developed or metastatic disease [7]. To date, studies have not reported a satisfactory solution to drug resistance [8]. Therefore, many patients have problems with chemo-resistance to paclitaxel. Identifying methods to overcome drug resistance remains a significant challenge.

Over 80% of NSCLCs show a higher aberrant expression level of Mucin1 (MUC1). MUC1 is a transmembrane heterodimeric protein that undergoes autocleavage into two subunits [9]. MUC1 consists of a complex of the extracellular N-terminal subunit (MUC1-N), which contains characteristic glycosylated tandem repeats of the mucin family, and the transmembrane MUC1 C-terminal subunit (MUC1-C), which is a single-pass membrane protein that creates interactions with receptor tyrosine kinases in the cell membrane [9, 10]. Interaction with the tyrosine kinase induces the activation of downstream signaling, such as the MEK→ ERK and PI3K → AKT pathways [11]. MUC1-C has a centrally important role in PTX resistance and stemness [2] Some studies have shown that targeting MUC1-C suppresses the downstream AKT and ERK pathways and inhibits tumorigenicity in NSCLC cells via the inhibition of EGFR activation [12]. MUC1-C promotes MYC expression selectively in lung cancer cells by activating the WNT/β-catenin pathway via directly binding to β-catenin and forming a complex on the cyclin D gene promoter [10]. Other studies have shown that inhibition of MUC1-C increases the level of E-cadherin with an associated reduction in vimentin expression. Loss of function or expression of E-cadherin leads to cancer cells entering the mesenchymal state and increases metastasis [13, 14].

BMI1 proto-oncogene, polycomb ring finger (BMI-1), which was originally isolated from mouse lymphomas, functions as an oncogene in several tumors [15]. Subsequent studies have shown that BMI-1 regulates the self-renewal and transformation of prostate cancer cells, as well as cell growth and metastasis in renal cancers [16, 17]. In particular, the expression of BMI-1 has effects on tumor size, poor differentiation, and distant metastasis in NSCLCs [18].

Recently, increased research attention has been paid towards microRNAs (miRNAs) as new post-transcriptional regulators of gene expression. miRNAs have the ability to regulate gene expression by directly targeting mRNAs [19]. miRNAs are small noncoding RNAs comprising approximately 19 to 22 nucleotides [20, 21]. MiRNAs affect diverse physiological processes, including development, differentiation, stem cell maintenance, and cell identity [22, 23]. miRNAs also regulate the expression of certain oncogenes and tumor suppressors in cancer [22, 23]. miRNAs are present in serum and blood cells, making them potential biomarkers for cancer screening and drug development [24]. Especially, miR-128 plays an important role as a regulator in prostate cancer cell differentiation by inhibiting BMI-1, which plays a role in epigenetic gene silencing and stem cell renewal [25]. Furthermore, miR-128 has anti-proliferative and anti-tumorigenic effects on diverse cancer types that show overexpression of BMI-1, including prostate cancer [25] and breast cancer [26]. However, to the best of our knowledge, there is no evidence supporting an association between miR-128 and MUC1-C and BMI-1 in paclitaxel-resistant lung cancer cells. Therefore, the present study aimed to investigate these associations.

The results demonstrated that overexpression of miR-128 inhibits MUC1-C and BMI-1 expression in paclitaxel-resistant lung cancer cells, thereby suppressing paclitaxel-resistant lung cancer.

## RESULTS

### A549/PTX CD133^+^ cells have similar characteristics to lung CSCs

**Figure 1:**
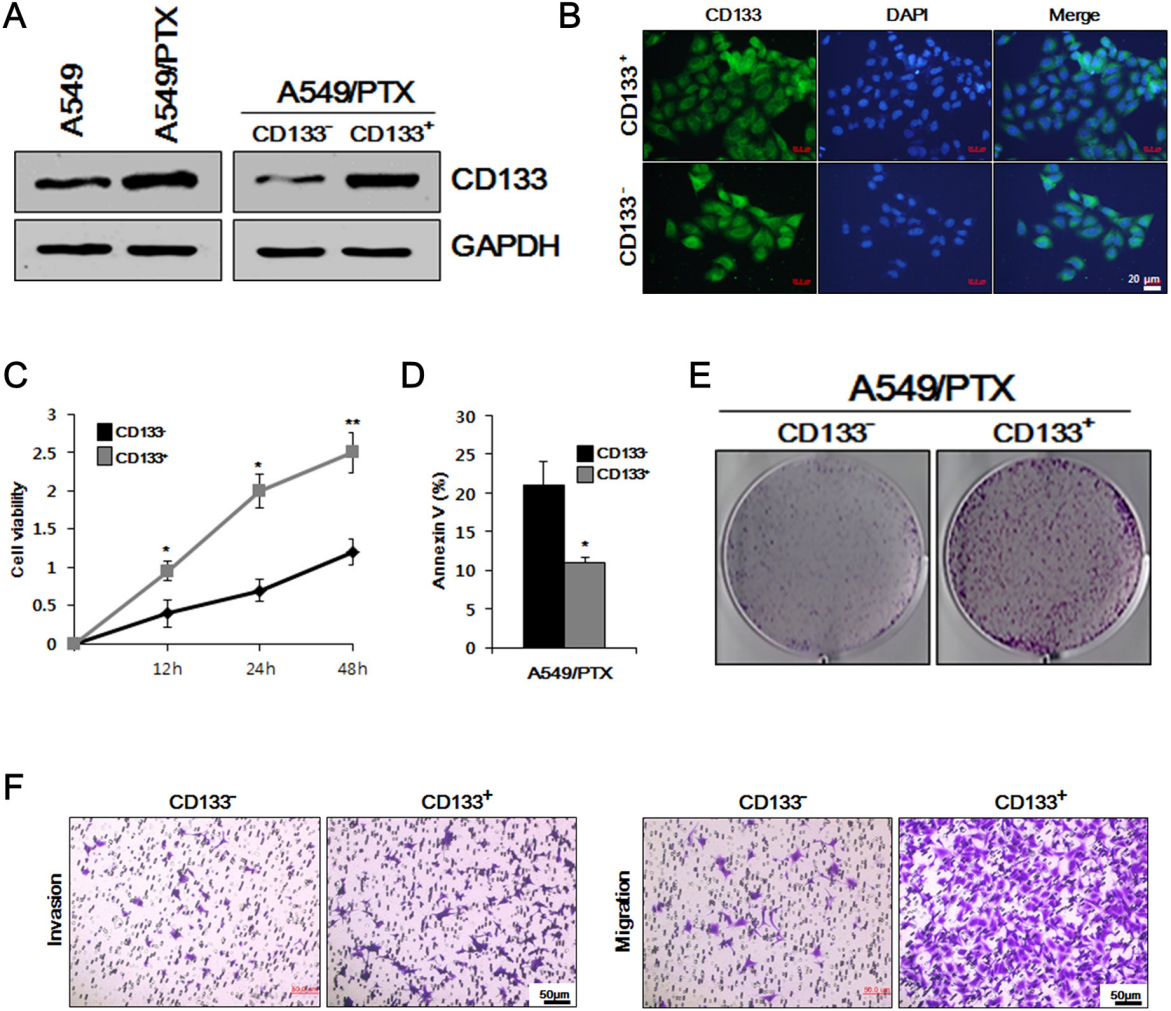
Comparative study of cell viability, proliferation, apoptosis, and metastasis in A549/PTX CD133+ and CD133- cells **(A)** Western blotting to determine the comparative levels of CSC surface marker CD133 in A549, A549/PTX, A549/paclitaxel (PTX) CD133^+^ and CD133^-^ cells. **(B)** Immunocytochemistry was performed to confirm the expression of CD133 in A549/PTX CD133^+^ and CD133^-^ cells. **(C)** Cell survival rates observed using a cell viability assay. **(D)** Apoptosis assay to analyze the cell death of CD133^+^ and CD133^-^ cells. **(E)** Colony formation assay to examine the cell proliferation in A549/PTX cells. **(F)** The metastatic ability of A549/PTX CD133^+^ and CD133^-^ cells investigate using migration and invasion assays.

Recent studies have reported that CD133 is a stem cell marker for both normal and cancerous stem cells [27]. We used western blotting to compare the level of CSC marker CD133 in A549 and A549/PTX cells, as well as in A549/PTX CD133^-^ and CD133^+^ cells. As shown in Figure 1A, the level of CD133 was higher in A549/PTX cells compared to that in A549 cells, while CD133^+^ cells showed higher levels of CD133 compared with CD133^-^ cells. These results indicated that the level of the CSC marker was comparatively higher in PTX-resistant lung cancer cells compared with that in normal lung cancer cells and was higher in CD133^+^ cells compared with that in CD133^-^ cells. To verify these results, we performed immunocytochemistry in A549/PTX CD133^+^ and CD133^-^ cells. As shown in Figure 1B, immunocytochemistry confirmed that the level of CD133 was higher in CD133^+^ cells compared with that in CD133^-^ cells. To observe the conditions of the cells, we performed cell viability and apoptosis assays on A549/PTX CD133^+^ and CD133^-^ cells. The cell viability assay assesses how healthy the cells are by measuring markers of cellular activity, and determines how well or how poorly the cells will respond to stress stimuli. The apoptosis assay determines the amount of cell death by measuring associated markers. As shown in Figure 1C and 1D, cell viability was higher in CD133^+^ cells compared with CD133^-^ cells, while cell death was higher in CD133^-^ cells compared with CD133^+^ cells. These results indicated that CD133^+^ cells are healthy and have higher cellular activities compared wth CD133^-^ cells, and that cell death is significantly lower than in CD133^-^ cells. To observe the cells’ proliferative and metastatic abilities, we performed colony formation, cell migration, and cell invasion assays on CD133^-^ and CD133^+^ cells. As shown in Figure 1E and 1F, the number of colonies and number of migrated and invaded cells were significantly higher in CD133^+^ cells compared with those in CD133^-^ cells, indicating higher cell proliferation and metastasis in CD133^+^ cells. These results suggested that the features of CD133^+^ cells show similarities with the characteristics of lung CSCs.

### A549/PTX CD133^+^ cells possess the characteristics of lung CSCs

**Figure 2:**
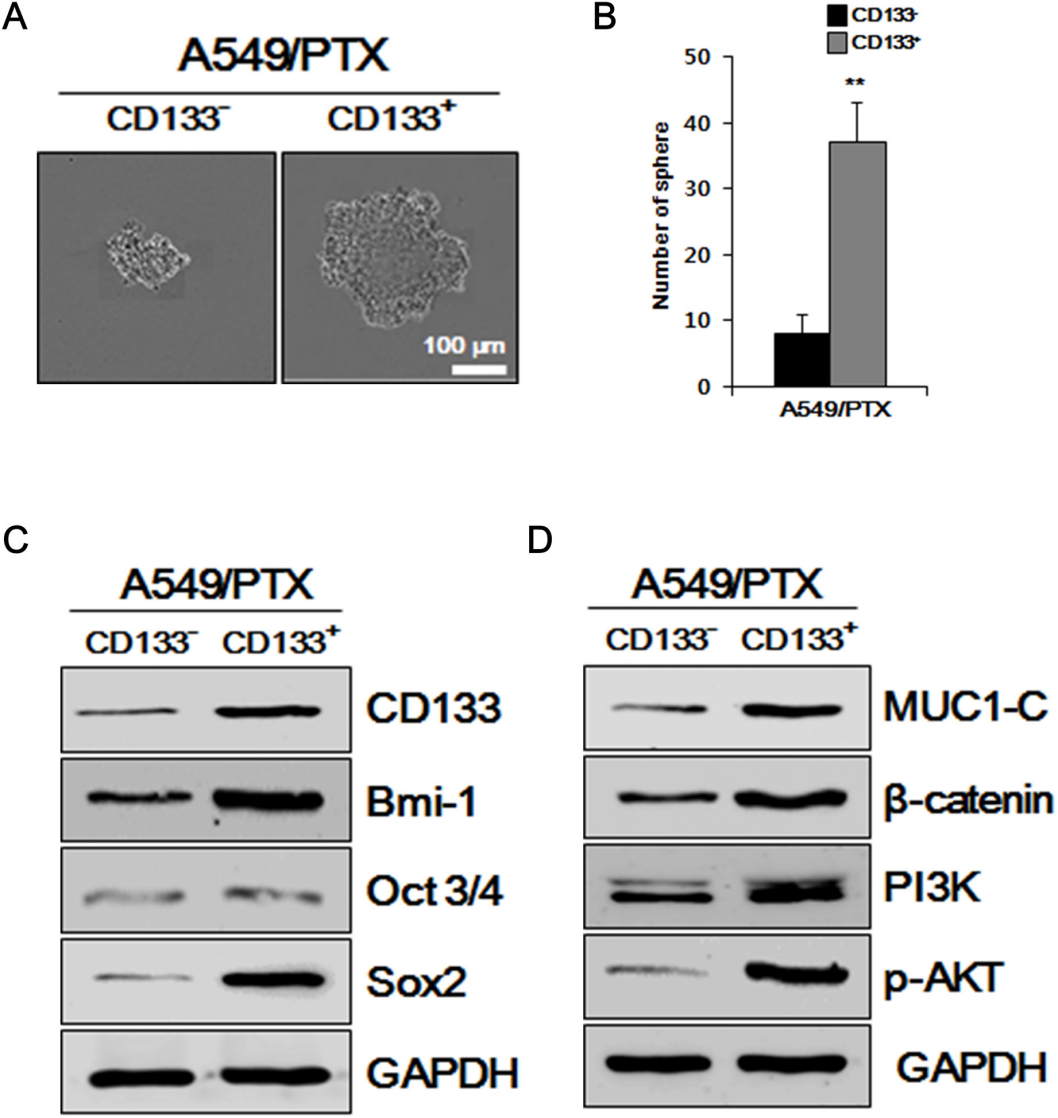
Examining A549/PTX cells for CSC characteristics **(A)** Sphere formation assay of A549/PTX CD133^+^ cells and CD133^-^ cells. **(B)** The number of spheres per well of A549/PTX CD133^+^ cells and CD133^-^ cells are presented. **(C)** Western blotting to determine the levels of BMI-1, cancer stem cell surface marker, stemness proteins, and **(D)** intracellular signaling pathways-related factors.

To determine the self-renewal ability of A549/PTX CD133^+^ cells and CD133^-^ cells, we performed sphere formation assays, which are performed to identify stem cells via their capacity of self-renewal and differentiation at the levels of single cells [28]. As shown in Figure 2A and 2B, the number and size of the spheres formed by CD133^+^ cells were significantly larger compared with those formed by CD133^-^ cells, indicating that A549/PTX CD133^+^ cells have a higher self-renewal capacity than CD133^-^ cells. To further examine whether the characteristics of A549/PTX CD133^+^ cells are related to the characteristics of CSCs, we performed western blotting analysis for stem cell markers, stemness-related proteins, and intracellular signaling pathway-related factors in A549/PTX CD133^+^ and CD133^-^ cells. As shown in Figure 2C, stem cell surface marker CD133 and stemness proteins OCT3/4, SOX2 expression levels were increased in A549/PTX CD133^+^ cells than in CD133^-^ cells, indicating that the cancer stem cell-marker and stemness protein levels are significantly higher in A549/PTX CD133^+^ cells than in CD133^-^ cells. As shown in Figure 2C and 2D, the levels of BMI-1 and MUC1-C were increased in A549/PTX CD133^+^ cells than in CD133^-^ cells. BMI-1 and MUC1-C are oncogenic proteins that interact with several signaling pathways related to self-renewal, cell proliferation, and cell migration [11, 29]; therefore, these data indicated higher self-renewal, proliferation, and migration capabilities of A549/PTX CD133^+^ cells. As shown in Figure 2D, expression levels of intracellular signaling pathways-related factors β-catenin, PI3K, and p-AKT were also increased in A549/PTX CD133^+^ cells compared with CD133^-^ cells. β-catenin is a proto-oncogene [30] that acts as an intracellular signal transducer in the WNT signaling pathway [31], thereby inducing stem cell renewal, epithelial-mesenchyme transition, cell-proliferation, and cell migration, and has a role in carcinogenesis [32, 33]. Kinases such as PI3K and AKT are involved in cellular functions, such as cell growth, proliferation, differentiation, motility, survival, intracellular trafficking and, thereby, cancer [34]. The endogenous levels of phosphorylated AKT were detected using anti-p-AKT antibodies. These results suggested that A549/PTX CD133^+^ cells show the characteristics of lung CSCs.

### Overexpression of miR-128 reduces the proliferation of A549/PTX CD133-positive cells *in vitro*

**Figure 3:**
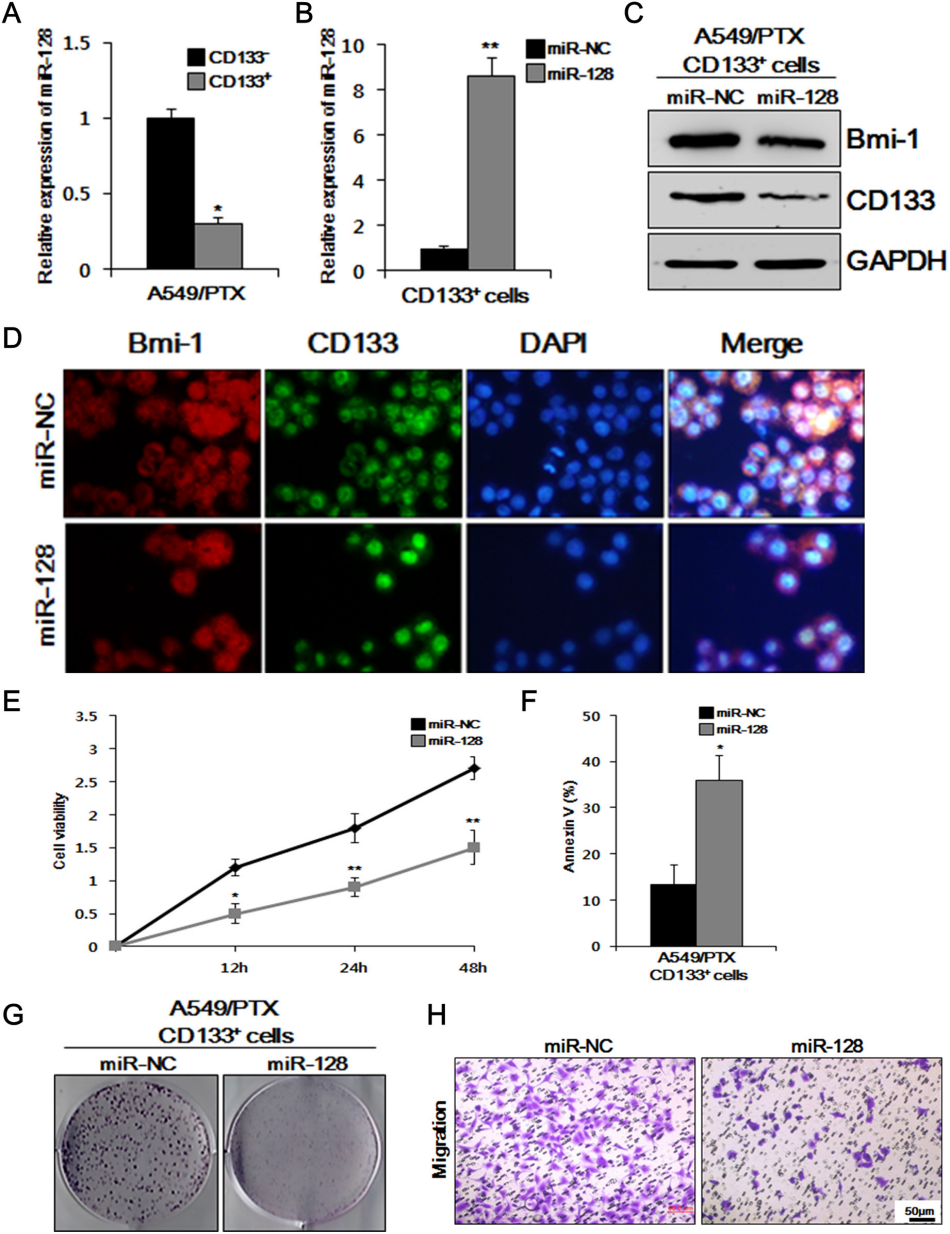
Overexpression of miR-128 inhibits cell viability, migration, colony formation, and BMI-1 expression, and induces apoptosis in CD133+ cells **(A)** Relative expression of miR-128 in A549/PTX CD133^+^ and CD133^-^ cells was measured by quantitative real-time reverse transcriptase-polymerase chain reaction (qRT-PCR) **(B)** and in A549/PTX CD133^+^ cells transfected with miR-128 and the negative control (NC). **(C)** Comparison of BMI-1 and CD133 levels between A549/PTX CD133^+^ cells treated with miR-128 and NC by western blotting. **(D)** Immunocytochemistry assay to verify the above comparative levels of BMI-1 and CD133. **(E)** Cell viability assay to assess the survival of the respective cells. **(F)** Apoptosis showing the effect miR-128 as treatment. **(G)** Colony formation assay to determine the cell proliferation ability of respective cells. **(H)** The migration capacity of A549/PTX CD133^+^ cells transfected with miR-128 and NC.

Although it has been reported that miR-128 downregulates BMI-1 in cancers [25, 26], miR-128-mediated downregulation of BMI-1 and reduction of the CSC-related characters in PTX-resistant lung CSCs has not yet been tested. Therefore, we transfected miR-128 and miR-NC, respectively, into the A549/PTX CD133^+^ cells and compared the effects of miR-128 and miR-NC on BMI-1 and CD133, and thereby, the lung CSC characteristics. We first observed the relative expression levels of miR-128 in A549/PTX CD133^-^ and CD133^+^ cells. There was little expression of miR-128 in CD133^-^ and CD133^+^ cells; therefore, the value of CD133- was fixed at 1 and compared with CD133^+^ cells. As shown in Figure 3A, the relative expression of miR-128 in CD133^+^ was lower than that in CD133^-^ cells. As shown in Figure 3B, the relative expression of miR-128 was higher in the cells transfected with miR-128 than in those transfected with miR-NC. To identify the relationship between miR-128 and oncogenic BMI-1 and CSC marker CD133, we performed western blotting analysis on A549/PTX CD133^+^ cells treated with miR-128 and miR-NC. As shown in Figure 3C, the levels of BMI-1 and CD133 were comparatively lower in A549/PTX CD133^+^ cells treated with miR-128 than in the cells treated with miR-NC. To visualize and verify the previous results, we conducted immunocytochemistry (ICC). As shown in Figure 3D, A549/PTX CD133^+^ cells treated with miR-128 showed significantly reduced cell growth and lower levels of BMI-1 and CD133 compared with the cells treated with miR-NC, indicating that miR-128 inhibits cell growth and reduces the levels of oncogenic proteins and CSC markers in PTX-resistant lung cancer stem cells. Moreover, to determine the cells condition, we performed cell viability and apoptosis assays on A549/PTX CD 133^+^ cells treated with miR-128 and miR-NC, respectively. As shown in Figure 3E, the condition of the A549/PTX CD133^+^ cells treated with miR-128 was significantly worse compared with that of the cells treated with miR-NC. As shown in Figure 3F, cell death was higher in cells treated with miR-128 compared with cells treated with miR-NC, indicating that miR-128 affects the health of cells and induces cell death in PTX-resistant lung cancer cells. To verify the effect of miR-128 on cell proliferation, we conducted colony forming assays on A549/PTX CD133^+^ cells. As shown in Figure 3G, the numbers of colonies formed by A549/PTX CD133^+^ cells treated with miR-NC were significantly higher than those formed by the cells treated with miR-128. To determine the effect of miR-128 on cell migration, we conducted a migration assay on A549/PTX CD133^+^ cells treated with miR-128 and miR-NC. As shown in Figure 3H, cells treated with miR-128 showed fewer migrated cells compared with the cells treated with miR-NC, indicating that miR-128 reduces the cell migration capacity. Taken together, these data indicated that overexpression of miR-128 inhibits BMI-1 expression and reduces CSC-related characteristics in PTX-resistant lung cancer stem cells.

### Overexpression of miR-128 downregulates CSC-related characteristics

**Figure 4:**
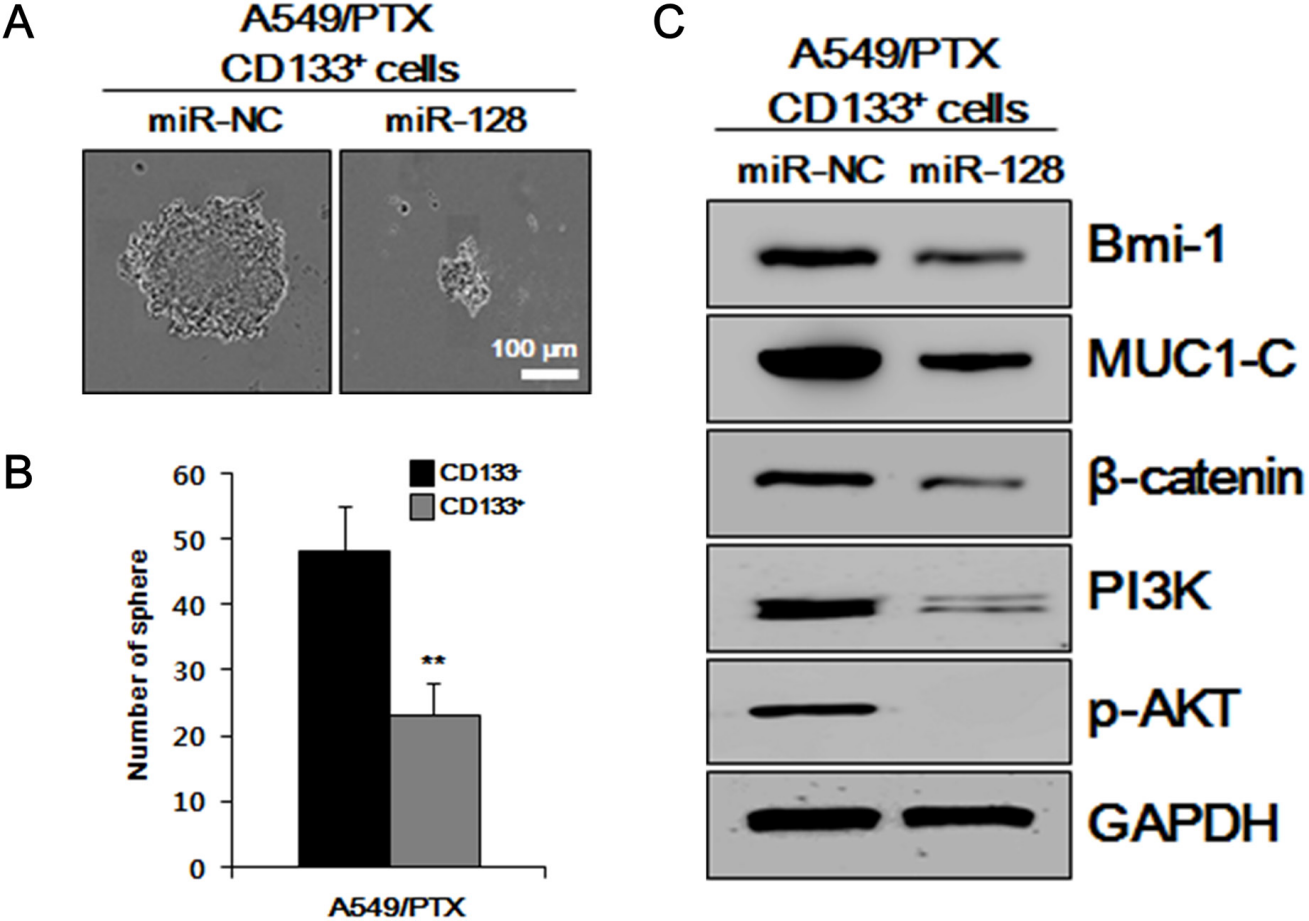
Effects of miR-128 overexpression *in vitro* on CSC-related characteristics **(A)** Results of a sphere formation assay performed on miR-NC-treated A549/PTX CD133^+^ cells and miR-128-treated A549/PTX CD133^+^ cells. **(B)** The numbers of spheres per well are presented. **(C)** Levels of intracellular signaling pathways-related factors, as determined by western blotting analysis.

To examine whether miR-128 is involved in the self-renewal of CSCs, the effects of miR-128 overexpression on sphere formation of A549/PTX CD133^+^ cells treated with miR-NC and miR-128 were determined *in vitro*. As shown in Figure 4A and 4B, overexpression of miR-128 significantly decreased the sphere formation and number of spheres of A549/PTX CD133^+^ cells, as analyzed by a sphere formation assay, indicating that miR-128 downregulates the self-renewal ability of CSCs. To further analyze the effect of overexpression of miR-128 on CSCs, western blotting analysis was performed on intracellular signaling pathways-related factors in A549/PTX CD133^+^ cells treated with miR-NC and miR-128. As shown in Figure 4C, the levels of intracellular signaling pathways-related factors were decreased in A549/PTX CD133^+^ cells treated with miR-128 compared with those in the cells treated with miR-NC. The PI3K→AKT intracellular signaling pathway is important to promote growth, proliferation over differentiation of stem cells, and cell cycle regulation, and is directly related to cancer [35]; therefore, these data indicated that overexpression of miR-128 downregulates CSC-related characteristics.

### MiR-128 inhibits the BMI-1 and cell growth by targeting MUC1-C in MUC1-overexpressing A549/PTX cells

**Figure 5:**
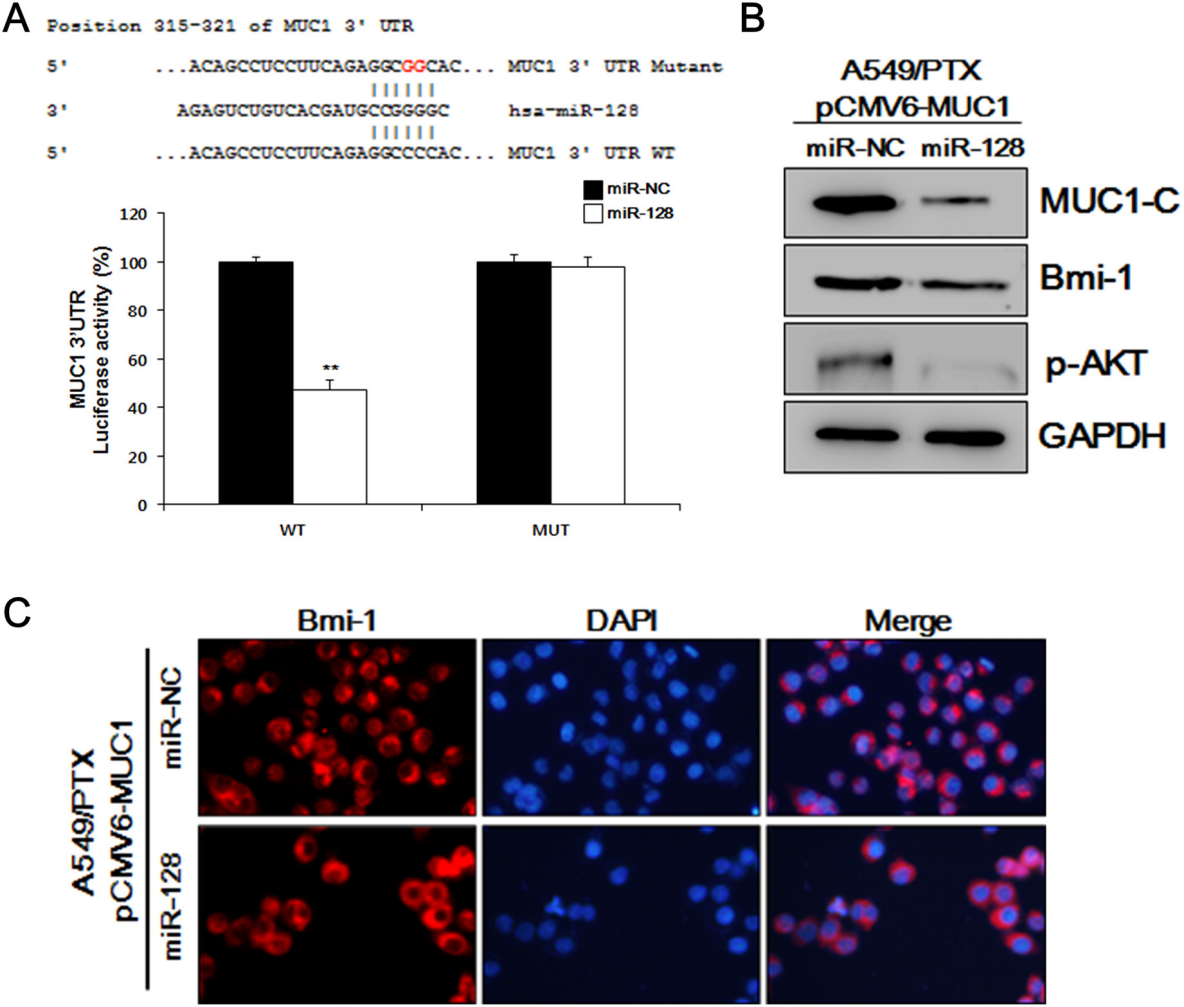
MUC1-C and BMI-1 are downstream targets of miR-128 **(A)** Mutated binding sequences of miR-128 in the *MUC1* 3′ UTR. Mutation was generated in the *MUC1* 3′ UTR by mutating 2 nucleotides that are recognized by miR-128. Either wild-type (WT) or mutant (MUT) MUC1 3′ UTR was subcloned into the dual-luciferase reporter vector. **(B)** Western blotting analysis of MUC1-C, BMI-1, and pAKT in A549/PTX pCMV6-MUC1 cells treated with miR-128. **(C)** Representative images of A549/PTX pCMV6-MUC1 cells treated with miR-128 and probed with an antibody against BMI-1.

Previously, we have shown specifically increased MUC1 levels in A549/PTX cells, and an activated AKT-related tumor growth mechanism [2]. Like other miRNAs, miR-128 may have multiple mechanisms contributing to tumor growth in A549/PTX cells. Here, we studied the correlation between MUC1 and miR-128 in A549/PTX. Using the bioinformatics prediction search (http://www.targetscan.org), we found that miR-128 targets the 3'-untranslated region (UTR) of a transcript variant of the *MUC1* mRNA. Although there is no published study confirming this relationship experimentally, this analysis results suggested a possible mechanism to support our hypothesis that *MUC1* expression is associated with the miR-128 level in A549/PTX pCMV6-MUC1 cells. To elucidate the molecular mechanisms by which miR-128 executes its function, we used a *MUC1* 3′ UTR luciferase reporter assay. As shown in Figure 5A, *MUC1* 3′ UTR luciferase reporter activity was reduced by miR-128 and that reduction was abolished by mutation of the *MUC1* 3′ UTR. Moreover, as shown in Figure 5B, transfection with miR-128 transcripts led to a reduction in transmembrane MUC1-C and stemness protein BMI-1 in A549/PTX pCMV6-MUC1 cells. As expected, in Figure 5C, miR-128 reduced the level of BMI-1 in A549/PTX pCMV6-MUC1 cells, as determined by ICC analysis. These data suggested that miR-128 inhibits CSC features by targeting *MUC1* expression.

### miR-128 inhibits tumor growth *in vivo*

**Figure 6:**
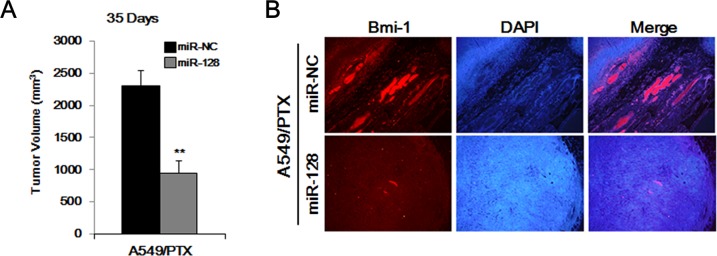
Overexpression of miR-128 inhibits the tumor-forming ability of A549/PTX CD133+ cells **(A)** Tumor formation of A549/PTX CD133^+^ cells treated with miR-128 and miR-NC. **(B)** Immunofluorescence of the tumor tissues from miR-128-treated mice and miR-NC-treated mice.

We used a mouse xenograft model to evaluate the *in vivo* effects of miR-128. A549/PTX cell tumors were established in nude mice, which were then divided into two groups (*n* = 5). As shown in Figure 6A, we observed larger sized tumors in the first group (treated with miR-NC) and smaller tumors in the miR-128-treated group. As shown in Figure 6B, we also found markedly decreased BMI-1 levels in tumors from mice that received miR-128 treatment compared with those in the miR-NC group, as determined by tissue immunofluorescence. These results indicated that miR-128 is a safe and effective therapy to treating PTX-resistant lung cancer.

## DISCUSSION

CSC properties have been reported in many human tumors and are thought to be responsible for tumor initiation, therapy resistance, progression, and metastasis [36]. CD133 is an important cell surface marker for the isolation of CSCs [37]. In addition, CDCs highly expressing CD133 have been shown to be invasive *in vitro* and are responsible for metastasis *in vivo* in mice [38].

In the current study, we first investigated the expression level of CSC marker CD133 in A549 and A549/PTX cells, as well as in A549/PTX CD133^-^ and CD133^+^ cells. The results showed that PTX-resistant A549 cells have higher levels of CD133, and that CD133^+^ cells have a higher level of CD133 compared with CD133^-^ cells. We further analyzed the malignant phenotypes of CD133^-^ and CD133^+^ cells. The results clearly demonstrated that CD133^+^ cells have a significantly more malignant phenotype, such as increased invasion, migration, colony formation, self-renewal, cell proliferation and lower apoptosis *in vitro*. Furthermore, our studies indicated that the levels of stemness proteins, oncogenic proteins, and intracellular signaling factors are higher in CD133^+^ cells compared with those in CD133^-^ cells.

MiRNAs that play critical roles in normal stem cell functions during development have emerged as important regulators of CSCs [36]. MiR-128 is associated with inhibition of CSC via targeting critical molecules such as BMI-1. BMI-1 is also indispensable for the regulation of self-renewal in non-small cell lung cancer. BMI-1 is highly enriched in CD133-positive cells of human glioblastoma multiforme [39]. A previous cell biology study revealed that BMI-1 prevents CD133-positive cell apoptosis and differentiation into neurons and astrocytes [39]. In addition, BMI-1 is involved in tumor growth and is required for CSC renewal and differentiation [39]. Overexpression of miR-128 significantly blocked CSC self-renewal by directly targeting *BMI-1* [23]. To further investigate the role of miR-128 in A549/PTX CD133^+^ cells, we transfected the cells with miR-128. CD133^+^ cells transfected with miR-NC were used as negative control. We found a significant downregulation of miR-128 in CD133^+^ cells compared with the CD133^-^ cells. Overexpression of miR-128 significantly increased the sensitivity of A549/PTX CD133^+^ cells to induce apoptosis. Furthermore, overexpression of miR-128 decreased the expression levels of stemness proteins, oncogenic proteins, and intracellular signaling factors. CSC feature such as cell proliferation, self-renewal ability, invasion, migration, and colony formation were also downregulated by the overexpression of miR-128 in CD133^+^ cells.

The PI3K/AKT signal transduction is involved in the regulation of multiple cellular functions including cell proliferation, survival, differentiation, adhesion, motility, and invasion [40]. PI3K signaling is closely related to MUC1 because MUC1-C can bind to PI3K and induce the PI3K/AKT pathway. This binding could be disrupted by an MUC1 inhibitor. In addition, when MUC1 is blocked, the rapamycin pathway, which is a target of PI3K, is downregulated [41]. In the present study, we found that miR-128 directly downregulated *MUC1* through its 3'-UTR in A549/PTX pCMv-MUC1 cells. *MUC1* represents a bona fide as well as functional target of miR-128, because expression of a *MUC1* cDNA lacking the miR-128 binding site at the 3'-UTR partly rescued the proliferation and sphere-forming defects in miR-128 overexpressing A549/PTX cells. These observations suggested that *MUC1* is an important downstream target for miR-128 to exert its tumor inhibitory functions.

Furthermore, overexpression of miR-128 in A549/PTX cells induced reduced PTX resistance, and MUC1-C, BMI-1, and PI3K signaling, suggesting that an increase in CSC properties is associated with PTX resistance and MUC1-C regulation in NSCLC. We confirmed that miR-128 overexpression decreased the protein levels of BMI-1 and MUC1-C in A459/PTX cells. MiR-128 abolishes chemo-resistance by inhibiting PI3K/AKT signaling [42]. Moreover, miR-128 can inhibit BMI-1, cancer stem cell proliferation, and xenograft growth *in vitro* and *in vivo*.

In conclusion, our results revealed that miR-128 induces apoptosis of paclitaxel-resistant lung cancer cells and decreases MUC1 and stemness-related protein BMI-1 levels. *MUC1* and *BMI1* are direct targets of miR-128 in paclitaxel-resistant lung cancer cells. Our results supported the hypothesis that miR-128 treatment is a potential therapeutic strategy for paclitaxel-resistant lung cancer by targeting MUC1-C and BMI-1.

## MATERIALS AND METHODS

### Cell culture

The PTX-resistant cancer cell line A549 was cultured in Roswell Park Memorial Institute (RPMI) 1640 (Invitrogen, US) containing 10% fetal bovine serum (FBS, Welgene, US) and 5 mL penicillin (Invitrogen, US). The cells were maintained in an incubator at 37°C with 5% CO_2_. Medium of CD133^+^ cells sorted from A549-PTX were supplemented with 10 ng/mL human epidermal growth factor (hEGF) (Sigma Aldrich, US) and 20 ng/mL of basic fibroblast growth factor (bFGF) (Koma Biotech, Daejeon, Korea).

### MTT 3-(4,5-dimethylthiazol-2-yl)-2,5-diphenyltetrazolium bromide) assay for cell viability

Cells (A549/PTX and miR-128 transfected cells) were seeded at 1 × 10^5^ cells/well in 96-well plates for 24 h. Thereafter, 10 μL of EZ-Cytox (Dogenbio, Korea) was added to each well, and the plates were incubated for an additional 1 h at 37°C. The optical density (OD) was the measured using a scanning multi-well spectrophotometer at a wavelength of 450 nm.

### Apoptosis assay

For the cell apoptosis analysis, A549/PTX and miR-128 transfected cells were seeded at 1 × 10^5^ cells/well in 6-well plates for 24 h. Thereafter, the cells were stained with fluorescein isothiocyanate (FITC)-conjugated Annexin V and Propidium lodide (PI). Data were collected and resolved using a FACS Calibur flow cytometer (BD Biosciences).

### Western blotting analysis

A549/PTX protein samples were loaded onto a gel for sodium dodecyl sulfate-polyacrylamide gel electrophoresis (SDS-PAGE), and run in 1 × Tris Glycine buffer (T&I, Korea). The proteins were transferred onto a nitrocellulose membrane (BIO-RAD, US). The membranes were incubated with primary antibodies against CD133 (Invitrogen), BMI-1 (Cell Signaling, USA), OCT3/4, SOX2, MUC1-C, β-catenin, PI3-K, p-AKT, and GAPDH (AbFrontier, Kroea) at 4°C overnight. Thereafter, the membranes were incubated with goat anti-rabbit IgG (Invitrogen, US) or mouse IgG (Santa Cruz Biotechnology, US) at 4°C overnight. Specific binding was detected using the SuperSignal chemiluminescent substrate (Thermo Fisher Scientific, US).

### Magnetic-activated cell sorting (MACS) separation of CD133 positive lung cancer stem cells

A549/PTX cells were labeled with a primary anti-CD133 antibody (Miltenyi Biotec, Germany) and subsequently separated on a MACS LS column. All procedures were carried out according to manufacturer's instructions. The purity of sorted cells was evaluated by western blotting.

### Immunocytochemistry assay

A549/PTX cells (5 × 10^4^ cells/well) were plated onto four-well plates in growth medium for 24 h. The cells were fixed in 3.7% formaldehyde for 10 min and incubated in blocking solution (1% bovine serum albumin (BSA)) for 1 h at room temperature. The cells were incubated with primary antibodies against CD133 and BMI-1 diluted in blocking buffer overnight at 4°C and then stained by a red fluorescent secondary antibody against rabbit IgG with PE, and a green fluorescent secondary antibody against rabbit IgG with Alexa Fluor488 for 2 hours. Nuclei were stained for 15 min with 2-(4-amidinophenyl)-1H-indole-6-carboxamidine (DAPI). The images were captured under an inverse microscope.

### Clonogenic assay

A549/PTX cells (1 × 10^3^ cells/well) were plated onto six-well plates in growth medium and maintained in an incubator at 37°C with 5% CO_2_ for 7 days. The cells were then washed with 1× phosphate-buffered saline (PBS), fixed with 3.7% formaldehyde for 10 min, treated with methanol for 20 min, and stained with crystal violet for 30 min. The plates were washed three times with 1× PBS.

### Sphere formation assay

A549/PTX cells (2 × 10^3^ cells/well) were plated respectively onto 6-well Ultra Low Cluster plate (Corning) and cultured in Dulbecco's modified Eagle's medium (DMEM)/F12 (Invitrogen, US) containing B27 supplement (1:50, Invitrogen, US), 10 ng/mL hEGF, 20 ng/mL bFGF, and 1% antibiotics (Invitrogen, US) for 10-14 days. After 10-14 days, the number of PTX cell spheres were counted, and images of the spheres were captured under an inverse microscope. Sphere formation efficiency = colonies/input cells × 100%

### Cell migration assay

The migration assay was determined using 24-well migration chambers (pore size 8 μm, Merck Millipore, Germany). The cells, at concentration of 1 × 10^5^ cells/well, were seeded in the migration chamber in 200 μL of RPMI1640 supplemented with 0.5% FBS; the lower chamber well contained 800 μL of RPMI1640 supplemented with 20% FBS to stimulate cell migration. After incubation for 24 h, non-invading cells were removed from the upper chamber, while the bottom cells were fixed with 3.7% paraformaldehyde and stained with 0.1% crystal. Cell migration was quantified by counting stained cells in five randomly selected fields at 100 × magnification under a light microscope.

### Cell invasion assay

The invasion assay was performed with A549/PTX cells using 24-well Matrigel invasion chambers. The cells were seeded in the upper chamber at a concentration of 7.5 × 10^4^ cells/well and incubated for 48 h. Then the cells in the invasion chamber were removed and fixed for counting. Cell invasion was quantified by counting stained cells in five randomly selected fields at 100 × magnification under a light microscope.

### Immunofluorescence

Paraffin-embedded tumor tissues were cut in 4-μm sections, deparaffinized in xylene, and rehydrated through graded ethanol. Sections were then rinsed three times in 1 × PBST (PBS with Tween 20). The sections were then blocked in 1 × PBS containing 0.5% Triton X – 100 and 5% sheep serum for 30 min at room temperature before overnight incubation at 4°C with a BMI-1 rabbit monoclonal antibody (D20B7 XP(R)). Sections were again rinsed three times in 1 × PBST. To detect primary antibodies, the sections were incubated in the dark at room temperature for 90 minutes in goat anti-rabbit IgG-PE (Santa Cruz biotechnology). Slides were counterstained with DAPI diluted in 1 × PBS in the dark for 20 min before visualization and image capturing using a microscope.

### Xenograft model

Mice were maintained and used for experiments according to Institutional Animal Care and Use Committee-approved protocols of Jeju National University (Jeju, Korea). Eight-week-old female athymic BALB/c nude mice were divided into two groups (n = 5) and injected subcutaneously with 1 × 10^5^ A549/PTX cells transfected with miR-128 and miR-NC, respectively. Tumor growth was assessed by measuring the length and width of the tumor mass with calipers every 3 days. Tumor volumes were calculated by the formula: Volume = shortest diameter^2^ × longest diameter/2).

### Statistical analysis

Data were analyzed using SPSS v.20.0.1 software (SPSS Inc., Chicago, IL, USA). Differences between groups were evaluated with the χ2 test or Fisher's exact test, as appropriate. P < 0.05 was considered statistically significant.

## Abbreviations

CSCs: cancer stem cells
SCLC: small cell lung cancer
NSCLC: non-small cell lung cancer
MUC1: Mucin1
MACS: Magnetic-activated cell sorting
ICC: immunocytochemistry
MUC1: Mucin1
MUC1-C: MUC1 C-terminal subunit
miRNAs: microRNAs
MTT: 3-(4,5-dimethylthiazol-2-yl)-2,5-diphenyltetrazolium bromide)
OD: optical density
